# Interaction of Agaric Acid with the Adenine Nucleotide Translocase Induces Mitochondrial Oxidative Stress

**DOI:** 10.1155/2020/5253108

**Published:** 2020-12-22

**Authors:** Edmundo Chávez, Mabel Buelna-Chontal, Arturo Macías-López, Luz Hernández-Esquivel, Francisco Correa, Natalia Pavón

**Affiliations:** ^1^Departamento de Bioquímica, Instituto Nacional de Cardiología, Ignacio Chávez Ciudad de México, Mexico; ^2^Departamento de Biomedicina Cardiovascular, Instituto Nacional de Cardiología, Ignacio Chávez Ciudad de México, Mexico; ^3^Departamento de Farmacología, Instituto Nacional de Cardiología, Ignacio Chávez Ciudad de México, Mexico

## Abstract

Mitochondrial permeability transition is characterized by the opening of a transmembranal pore that switches membrane permeability from specific to nonspecific. This structure allows the free traffic of ions, metabolites, and water across the mitochondrial inner membrane. The opening of the permeability transition pore is triggered by oxidative stress along with calcium overload. In this work, we explored if oxidative stress is a consequence, rather than an effector of the pore opening, by evaluating the interaction of agaric acid with the adenine nucleotide translocase, a structural component of the permeability transition pore. We found that agaric acid induces transition pore opening, increases the generation of oxygen-derived reactive species, augments the oxidation of unsaturated fatty acids in the membrane, and promotes the detachment of cytochrome c from the inner membrane. The effect of agaric acid was inhibited by the antioxidant tamoxifen in association with decreased binding of the thiol reagent eosin-3 maleimide to the adenine nucleotide translocase. We conclude that agaric acid promotes the opening of the pore, increasing ROS production that exerts oxidative modification of critical thiols in the adenine nucleotide translocase.

## 1. Introduction

An essential mitochondrial function is to provide the cell with the energetic requirements for its metabolic processes. To accomplish this, a selectively permeable membrane is required to guarantee the establishment of a proton gradient across the inner membrane. The nonselective permeability process, widely known as mitochondrial permeability transition (mPT), disrupts the proton gradient that powers oxidative phosphorylation [1]. The nonspecific pore has a diameter of up to 3 nm and allows the free traffic of ions, metabolites, and even DNA fragments [2]. The molecular nature of this nonspecific pore has been the matter of debate for the last 30 years and is still under debate. Early experiments pointed to adenine nucleotide translocase (ANT) [3, 4] or to the phosphate carrier [5, 6] as the main structure confirming the mitochondrial permeability transition pore (mPTP). Recently, the interphase between *c* subunits of the F_1_-ATP synthase dimers has been pointed out as the nonspecific pore, and in this new model, ANT plays a regulatory role [7]. The pore is opened by a large number of inducers, including carboxyatractyloside [8], Ca^2+^ [9], thiol-blocking reagents, heavy metals [10–13], and oxidative stress. It is known that oxidative stress is a main factor that promotes mPTP opening in an isolated mitochondria [9, 14], in intact cells [15] and during reperfusion damage [16]. However, it is not clear if reactive oxygen species (ROS) generation is a consequence of pore opening, or if endogenously generated ROS accounts for its aperture. Thus, the objective of this work was to explore the possibility that the opening of the nonspecific pore may induce oxidative stress. We used agaric acid (Ag Ac) to induce the aperture of the pore, as we previously reported its interaction with ANT [17]. The experimental results show that Ag Ac caused pore opening, resulting in drop of the transmembrane electric gradient (ΔΨ). The collapse of ΔΨ amplified H_2_O_2_ production, increased the oxidation of the lipid milieu of the inner membrane, and inhibited the activity of superoxide dismutase. ROS increase might result from detachment of cytochrome c from the inner membrane. According to these results, it is provocative to assume that the opening of the nonspecific pore promoted oxidative stress, as such an event appears to be inhibited by the oxidant molecule tamoxifen (TAM).

## 2. Materials and Methods

Chemicals were of reagent or higher grade from Sigma-Aldrich (St Louis, MO) unless otherwise specified. Anticytochrome c rabbit monoclonal antibody (Ab76237) was from Abcam (Cambridge, MA, USA), and anti-VDAC mouse monoclonal was (sc390996) from Santa Cruz Biotechnology (CA, USA). The enhanced chemiluminescence detection system was from Millipore Corporation (Bedford, MA, USA), and horseradish peroxidase- (HRP-) conjugated secondary antibodies were from Abcam (Cambridge, MA, USA).

The investigation was approved by the Ethics Committee of the National Institute of Cardiology, “Ignacio Chávez” (INC-13806), and the experimental protocols followed the guidelines of Norma Oficial Mexicana for the use and care of laboratory animals (NOM-062-ZOO-1999) and for disposal of biological residues (NOM-087-SEMARNAT-SSA1-2002).

### 2.1. Mitochondrial Protein

The mitochondria from the rat kidney cortex were prepared by homogenization of the tissue in 0.25 M sucrose 1 mM EDTA, 10 mM TRIS adjusted with Tris to pH 7.3, following standard centrifugation procedures. Protein content was determined following the method of Lowry et al. [18].

### 2.2. Ca^2+^ Retention Capacity, Mitochondrial Swelling, and Transmembrane Gradient Measure

The experiments were carried out by incubating 2 mg of mitochondrial protein in 3 ml of basic medium containing 125 mM KCl, 10 mM succinate, 3 mM phosphate, 10 mM HEPES, 5 *μ*g rotenone, and 2 *μ*g oligomycin. The medium was adjusted to pH 7.3. The movements of Ca^2+^ were assayed spectrophotometrically at 675–685 nm using the metallochromic indicator Arsenazo III. Mitochondrial swelling was followed at 540 nm. The transmembrane electric gradient (ΔΨ) was determined using the dye Safranine.

### 2.3. Superoxide Dismutase (SOD) Activity

The activity of the enzyme superoxide dismutase was analyzed in the mitochondria by nondenaturating acrylamide gel electrophoresis as described by Pérez-Torres et al. [19]. Briefly, it used 100 micrograms of mitochondrial protein in a nondenaturing gel electrophoresis and nitro blue tetrazolium (NBT). The electrophoresis was carried out at 100 v for 5 h. At the end, the gel was incubated in 2.45 mM NBT for 30 min, the liquid was discarded, and the gel was incubated in a 28 mM TEMED solution containing 36 mM potassium phosphate (pH 7.8) and 0,028 mM riboflavin in the dark for 10 min. The blue NBT stain was developed by exposure to UV light for another 10 min. A standard curve was obtained with a serial dilution (2.5, 5, 10, 15, 30, and 60 ng) of SOD from bovine erythrocytes.

### 2.4. Lipid Peroxidation and H_2_O_2_ Content

Lipid peroxidation of the mitochondrial membrane was carried out with the thiobarbituric acid reactive substances using tetraethoxypropane as standard as previously described [13]. H_2_O_2_ content was evaluated according to the procedure of Dikalov et al. [20], by incubating 2 mg of mitochondrial protein in 3 ml of a basic medium and 10 *μ*mol L^−1^ 10 acetyl-3,2-dihydrophenoxazine plus 0.2 U/ml horseradish peroxidase in a dark chamber at 37°C for 60 min. The product, resorufin, was determined based on an increase in fluorescence at 530–590 nm.

### 2.5. Mitochondrial DNA and Cytochrome C Content

Mitochondrial DNA was obtained as reported previously [2]; its disruption was resolved in 0.8% agarose gels and visualized with ethidium bromide under UV light.

### 2.6. The Content of Mitochondrial Cytochrome C

The content of cytochrome C was analyzed by loading 50 *μ*g of protein from the mitochondria onto 15% SDS-PAGE gel and transferred to PVDF membranes for immunodetection. A primary monoclonal antibody against cytochrome C and a secondary HRP-conjugated antibody were used to evaluate the protein content of the membrane. The content of the voltage-dependent anion channel (VDAC) was used as loading control.

### 2.7. Adenine Nucleotide Translocase Detection

Adenine nucleotide translocase was labeled as previously reported by Majima et al. [21]; 1 mg of mitochondrial protein was suspended in 1 ml of a basic medium and preincubated with 3 *μ*M agaric acid and 20 *μ*M tamoxifen for 5 min; subsequently, 20 nmol/mg eosin maleimide (EMA) was added and incubated for 5 min at 4°C in darkness. The reaction was stopped by adding 30 mM DTT(dithiothreitol). Then, 300 *µ*g of mitochondrial protein was then subjected to SDS-PAGE in 10% polyacrylamide under nonreducing conditions. Fluorescence was detected with a UV-lamp.

## 3. Results

**Figure 1:**
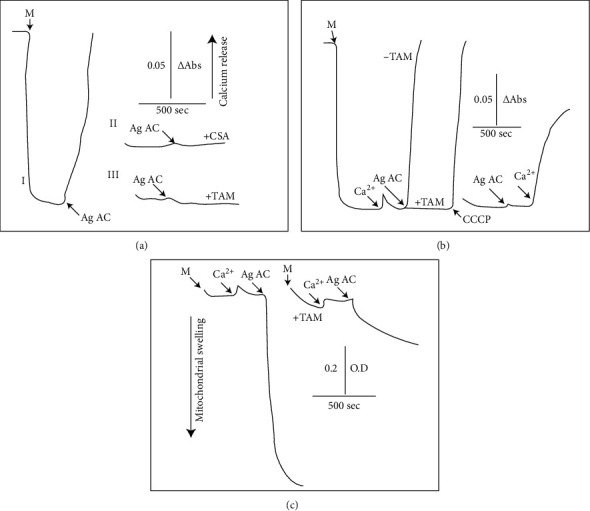
(a) Agaric acid effect on the matrix Ca^2+^ content release. Mitochondrial protein (2 mg) was added to 3 ml of a basic medium as described in Materials and Methods. Where indicated, 3 *μ*M agaric acid (Ag Ac), 2 *µ*M cyclosporin A (CSA), and 20 *µ*M tamoxifen (TAM) were added. Traces are representative of, at least, 4 different experiments. (b) Agaric acid-induced collapse of the transmembrane electric gradient. Mitochondrial protein was incubated in 3 ml of a medium. Where indicated, 50 *µ*M CaCl_2_, 3 *µ*M agaric acid, 20 *µ*M tamoxifen, and 1 *µ*M of the uncoupler CCCP were added. (c) Attenuation by tamoxifen of the agaric acid-induced mitochondrial swelling. Mitochondrial protein was incubated in 3 ml of basic. Where indicated, 50 *µ*M CaCl_2_, 3 *µ*M agaric acid, and 20 *µ*M tamoxifen were added. Temperature: 25°C. Traces are representative of, at least, 4 different experiments.

Figure 1(a)) shows the inhibitory effect of tamoxifen on the efflux of mitochondrial Ca^2+^ as induced by Ag Ac. Calcium was accumulated into mitochondria oxidizing succinate and reached a steady state until the addition of 3 *μ*M Ag Ac that initiated a fast release of this cation (I). This was completely abolished by cyclosporin A (CSA) (II), indicating the mediation of the permeability transition pore. In (III), it is shown that 20 *μ*M of the antioxidant tamoxifen (TAM) also inhibited matrix Ca^2+^ efflux induced by Ag Ac.

Building up of the transmembrane electric gradient is an essential prerequisite to sustain oxidative phosphorylation. In Figure 1(b), it is shown that Ca^2+^ exerts temporal depolarization that is corrected when the rates of uptake and release reach the equilibrium. The addition of 3 *μ*M Ag Ac exerted a fast drop of membrane potential; however, this feature of the permeability transition was eliminated when the medium was supplemented with TAM. It is also shown that the uncoupler carbonyl cyanide m-chlorophenylhydrazone (CCCP) dissipates the transmembrane electric gradient and that that Ca^2+^ is required to promote the collapse of ΔΨ induced by Ag Ac (right panel).

Mitochondrial swelling also characterizes the permeability transition pore opening. The experimental results presented in Figure 1(c)) show that Ag Ac induced a fast and extensive swelling, which was inhibited after the addition of TAM.

**Figure 2:**
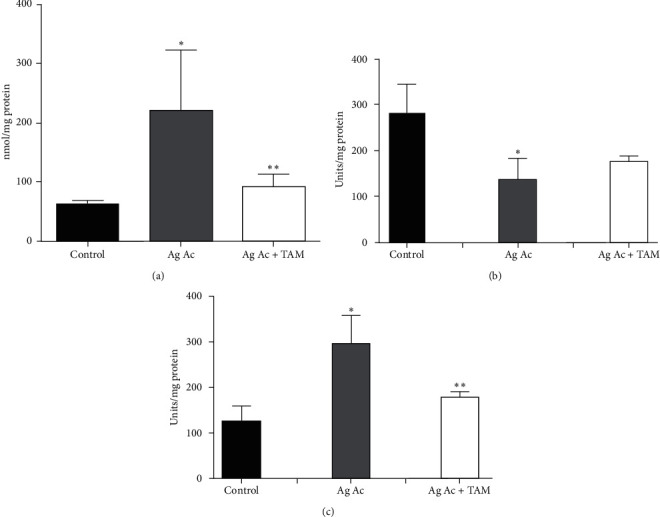
(a). Increased generation of hydrogen peroxide by agaric acid and its inhibition by tamoxifen. Mitochondrial protein (2 mg) was incubated in 3 ml of a basic medium. Where indicated, 3 *µ*M agaric acid (Ag Ac) and 20 *µ*M tamoxifen were added. Results are the mean ± SD of 3 different experiments. ^*∗*^*p* < 0.05 vs. control; ^*∗∗*^*p* < 0.05 vs. Ag Ac. (b). Protective effect of tamoxifen on the agaric acid-induced inhibition of the superoxide dismutase enzyme. The added concentrations of agaric acid (Ag Ac) and tamoxifen were 3 *µ*M and 20 *µ*M, respectively. The data represent the mean ± SD of six different experiments. ^*∗*^*p* < 0.05 vs. control; ^*∗∗*^*p* < 0.05 vs. Ag Ac. (c). Agaric acid-induced oxidation of the membrane lipid milieu and the inhibitory effect of tamoxifen. Mitochondrial protein (2 mg) was added to 3 ml of a basic medium. Where indicated, 3 *µ*M agaric acid (Ag Ac) and 20 *µ*M tamoxifen (TAM) were added. The data represent the average of six different experiments ± SD of six different experiments. ^*∗*^*p* < 0.05 vs. control; ^*∗∗*^*p* < 0.05 vs. Ag Ac. Incubation temperature: 25°C.

Next, we explore the possibility that Ag Ac induces oxidative stress by measuring the generation of hydrogen peroxide. Ag Ac did augment the production of H_2_O_2_ (Figure 2(a)), and such rise appears to be inhibited after the addition of TAM.

Mitochondrial damage resulting from increased H_2_O_2_ levels would normally be ameliorated via the activity of the first line member of the mitochondrial antioxidant system, i.e., superoxide dismutase enzyme (SOD). As observed in Figure 2(b)), Ag Ac inhibited SOD activity by 50%, and such inhibition was partially reversed in Tam-treated mitochondria.

**Figure 3:**
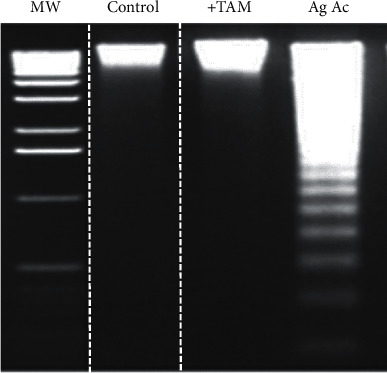
Effect of agaric acid on mitochondrial DNA disruption and its protection by the antioxidant tamoxifen. Experimental conditions were as described under Materials and Methods. Where indicated, 3 *µ*M agaric acid and 20 *µ*M tamoxifen (+Tam) were added. The image is representative of 3 different experiments.

Increased oxidative stress, resulting from augmented hydrogen peroxide levels and diminished SOD activity, promoted damage to lipids, DNA, and proteins in the mitochondria. The oxidation of membrane polyunsaturated fatty acids was evaluated measuring the levels of thiobarbituric acid reactive substances (TBARS), which are produced as byproducts of lipid peroxidation. As observed, Tam diminished the oxidation of membrane fatty acids induced by Ag Ac (Figure 2(c)). Oxidative stress also induces damage on mitochondrial DNA (mtDNA).  Figure 3 shows that Ag Ac induces the rupture of the genetic material. As is illustrated, mtDNA degradation was prevented by the antioxidant TAM.

**Figure 4:**
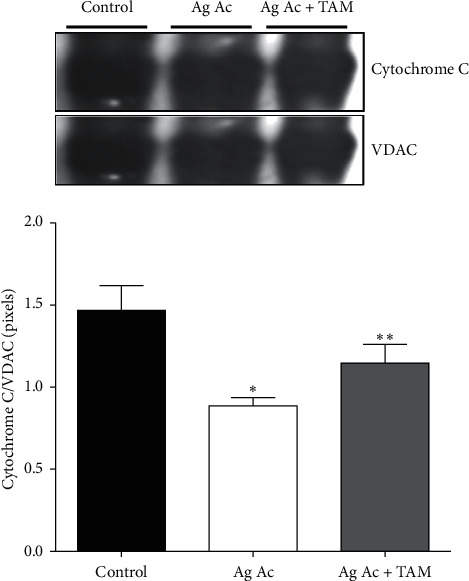
Agaric acid-induced detachment of cytochrome c and its protection by tamoxifen. Experimental conditions were as described in Materials and Methods. Data represent mean ± SD of, at least, three different experiments ^*∗*^*p* < 0.05 vs. control; ^*∗∗*^*p* < 0.05 vs. Ag Ac. The image is representative of, at least, 3 different experiments.

It is known that mitochondrial permeability transition might contribute to apoptosis by releasing cytochrome c from the mitochondria [22, 23]. In this context, we found that Ag Ac promoted the detachment of this protein from the inner membrane. As can be seen from Figure 4, cytochrome c content diminished by 40% in Ag Ac-treated mitochondria, and this decrement was partially abolished by TAM.

**Figure 5:**
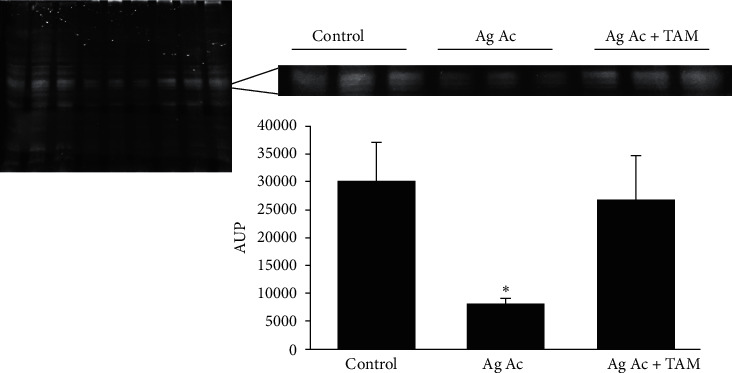
Effect of tamoxifen on the diminution of EMA binding to the adenine nucleotide translocase. Experimental conditions were as indicated in Materials and Methods. Where indicated, 3 *µ*M agaric acid (Ag Ac) and 20 *µ*M tamoxifen (TAM) were added.

To determine if oxidative stress induced by Ag-Ac impacted on ANT, we labeled the mitochondria with the fluorescent probe eosin-5-maleimide (EMA). According to Majima et al. [21], this compound binds to Cys159 on the adenine nucleotide translocase, which has a molecular weight of 30 kDA. It is shown in Figure 5 that Ag Ac reduced the fluorescent labeling of mitochondrial proteins by EMA, in particular of proteins weighting 30 kDa (lines 4–6) in comparison with control mitochondria (lines 1–3). The addition of tamoxifen restored the fluorescent mark (lines 7–9), indicating either that tamoxifen prevents the binding of Ag Ac near the Cys159 locus or that by diminishing ROS production, it prevented the oxidative modification of critical thiols in the adenine nucleotide translocase.

## 4. Discussion

Membrane pore permeability is an important biological phenomenon playing an essential role in normal biological processes [24], and its dysregulation turned out to be a crucial step in the pathogenesis of a variety of diverse diseases, encompassing ischemia-reperfusion damage [25], liver damage [26], many chronic and acute disorders of the central nervous system [27], and collagen VI myopathies [28].

Reports pointed out to ANT as a structural component of the nonspecific pore [3,4]. More recently, it has been proposed that the *c* subunit of the F1-ATP synthase is the main mPTP constituent and that ANT plays a regulatory role in its open-closed state [7]. The pore opening is triggered under different experimental settings, i.e., by adding thiol blocking reagents [10] and heavy metals [11–13, 29] or by inhibiting the mitochondrial respiratory chain [30, 31], and also in physiopathological conditions such as ischemia/reperfusion damage [16]. Oxidative stress and mPTP opening are “hallmark” of the injury developed during reperfusion [9, 32, 33]. As antioxidants prevent from the aperture of the mPTP, it is accepted that the sudden generation of ROS derives in the aperture of the nonspecific pore. Contrary to such view, recent reports have claimed that the opening of the nonspecific pore is the cause of oxidative stress [34]. In this work, we add evidence to this possibility by using Ag Ac to induce the aperture of the nonspecific pore and Tam, a molecule with antioxidant properties [35]. ROS generation took place after the addition of Ag Ac, along with diminished SOD activity, oxidation of membrane unsaturated fatty acids, and release of cytochrome c from the mitochondria. All these events were inhibited by the oxidant TAM.

There are reports showing that cytochrome c is released either before or after PT pore opening depending on the stimuli [36]. In the first scenario, cytochrome c release would increase ROS generation after complex III inhibition [9] promoting PT pore opening, which, in turn, might lead to more cytochrome c release and to further increase of ROS levels in a self-amplifying loop [37]. Another possible mechanism by which oxidative stress might be produced after the opening of a nonspecific pore is the close relationship between the transmembrane electric gradient and the generation of ROS [38]. Our experiments pointed out to this hypothesis, as agaric acid induced both a drop of ΔΨ, as well as the generation of H_2_O_2_.

Regarding the role of ANT in mPTP opening, we previously reported that Ag Ac might interact with the adenine nucleotide translocase [17] and also that Tam inhibits permeability transition as induced by carboxyatractyloside [39]. In this sense, an additional result that deserves special attention is that Tam prevented from the Ag Ac-induced decrease in EMA binding to ANT that might account for the protective effect of the antioxidant. It is known that Cys159 is the target site of EMA on adenine nucleotide translocase [21].

## 5. Conclusions

We propose that Ag Ac might interact with ANT through the cationic environment of a loop formed by Lys145, Arg151, Lys162, Lys165, and Arg170. This interaction might be reinforced through the interaction of the alkyl chain of Ag Ac with the hydrophobic milieu of the inner membrane. The hypothesis that such interaction induces pore opening is sustained by a previous publication [17], as well as by the results of Figure 1 in this work. Therefore, Tam reduces oxidative stress, either by preventing the binding of Ag Ac near the Cys159 locus or by acting as an antioxidant and preventing the oxidative modification of critical thiols in the adenine nucleotide translocase.

## Data Availability

Data used to support the findings of this study are given in the manuscript.
